# Assessment of Ligament Viscoelastic Properties Using Raman Spectroscopy

**DOI:** 10.1007/s10439-022-02988-z

**Published:** 2022-07-08

**Authors:** Andy Cui, Ervin Nippolainen, Rubina Shaikh, Jari Torniainen, Aapo Ristaniemi, Mikko Finnilä, Rami K. Korhonen, Simo Saarakkala, Walter Herzog, Juha Töyräs, Isaac O. Afara

**Affiliations:** 1 School of Information Technology and Electrical Engineering, The University of Queensland, Brisbane, Australia; 2 Department of Applied Physics, University of Eastern Finland, Kuopio, Finland; 3 Department of Orthopedics, Traumatology and Hand Surgery, Kuopio University Hospital, Kuopio, Finland; 4 Research Unit of Medical Imaging, Physics and Technology, Faculty of Medicine, University of Oulu, Oulu, Finland; 5 Department of Diagnostic Radiology, Oulu University Hospital, Oulu, Finland; 6 Science Service Center, Kuopio University Hospital, Kuopio, Finland; 7 AO Research Institute Davos, Davos, Switzerland; 8 Human Performance Lab, Faculty of Kinesiology, University of Calgary, Calgary, Canada

**Keywords:** Ligament, Raman spectroscopy, Mechanical properties, Viscoelastic properties

## Abstract

Injuries to the ligaments of the knee commonly impact vulnerable and physically active individuals. These injuries can lead to the development of degenerative diseases such as post-traumatic osteoarthritis (PTOA). Non-invasive optical modalities, such as infrared and Raman spectroscopy, provide means for quantitative evaluation of knee joint tissues and have been proposed as potential quantitative diagnostic tools for arthroscopy. In this study, we evaluate Raman spectroscopy as a viable tool for estimating functional properties of collateral ligaments. Artificial trauma was induced by anterior cruciate ligament transection (ACLT) in the left or right knee joint of skeletally mature New Zealand rabbits. The corresponding contralateral (CL) samples were extracted from healthy unoperated joints along with a separate group of control (CNTRL) animals. The rabbits were sacrificed at 8 weeks after ACLT. The ligaments were then harvested and measured using Raman spectroscopy. A uniaxial tensile stress-relaxation testing protocol was adopted for determining several biomechanical properties of the samples. Partial least squares (PLS) regression models were then employed to correlate the spectral data with the biomechanical properties. Results show that the capacity of Raman spectroscopy for estimating the biomechanical properties of the ligament samples varies depending on the target property, with prediction error ranging from 15.78% for tissue cross-sectional area to 30.39% for stiffness. The hysteresis under cyclic loading at 2 Hz (RMSE = 6.22%, Normalized RMSE = 22.24%) can be accurately estimated from the Raman data which describes the viscous damping properties of the tissue. We conclude that Raman spectroscopy has the potential for non-destructively estimating ligament biomechanical properties in health and disease, thus enhancing the diagnostic value of optical arthroscopic evaluations of ligament integrity.

## Introduction

Ligaments are viscoelastic bands of connective tissue that play a crucial role in the health, stability and mobility of the knee joint. Structural support of the knee is mediated by the distribution of mechanical loads between articular cartilage, ligaments and menisci.^30^ Injurious impact loading of the knee can potentially lead to post-traumatic osteoarthritis (PTOA) or other chronic joint conditions. Approximately 900,000 acute knee injuries are reported each year in the US.^27^ The consequences of ligament ruptures and other debilitating injuries to the anterior cruciate ligament (ACL), for instance, can result in PTOA-related cartilage lesions,^5,29^ bone bruises^17^ and meniscal tears.^8,18^ Furthermore, the biomechanical properties of knee ligaments other than the ACL may provide several reliable metrics that could aid in the evaluation of the severity of knee injuries.^28^ However, there are currently no techniques that can accurately measure ligament properties non-destructively during arthroscopic repair procedures.

Arthroscopy is the most reliable diagnostic approach for evaluating and treating joint injuries.^16^ However, conventional arthroscopy lacks the ability to facilitate a comprehensive and objective assessment of tissue integrity and mechanical properties.^25,26^ The implementation of a quantitative arthroscopic method would improve the diagnostic capability of traditional arthroscopy. In recent years, Raman spectroscopy has emerged as a viable tool for the study of OA and joint health.^19^ This is due to its sensitivity to the changes in composition of diseased joint tissue,^24^ even in early disease stages, and its suitability for *in vivo* assessment. While spectroscopic evaluation of the biomechanical properties of different knee tissues has been conducted previously,^1,20,28^ Raman spectroscopy has not been thoroughly tested, particularly on ligaments. The limitations of previously tested spectroscopic methods motivated this investigation into Raman spectroscopy for ligament evaluation. Water is typically the dominant constituent of biological tissue and significantly affects the resulting spectrum when infrared spectroscopy methods are employed. This often requires significant preprocessing and would require careful data collection for *in vivo* arthroscopy applications. Raman spectroscopy, on the other hand, is much less sensitive to water^10^ in comparison to infrared spectroscopic techniques.

Since knee ligaments function primarily in tension, tensile testing can characterise the viscoelastic and quasi-static material properties of these tissues. However, tensile testing is destructive as testing is typically continued until tissue failure. Furthermore, biological tissue is inherently inhomogeneous^10^ and as such, quantitative surgical methods capable of measuring the unique biomechanical properties for any individual joint can greatly improve the outcome of arthroscopic surgery. Therefore, a technique capable of providing orthopaedic surgeons with an accurate assessment of the structural integrity and overall condition of the knee joint may be of great benefit in the planning of arthroscopic repair surgery and the detection of early-stage PTOA.

This investigation was largely motivated by current limitations of ligament replacement surgery which involves the use of healthy ligaments from other parts of the body to replace damaged ligaments^12^ (e.g., replacing a torn ACL with a graft from the patellar tendon). The exact biomechanical properties of these two different tissues cannot be measured without invasive mechanical testing. Therefore, the implementation of optical spectroscopy in ligament repair surgery may provide orthopaedic surgeons with a reasonable estimate of the biomechanical properties of healthy and damaged ligaments.

In this study, an investigation into a Raman spectroscopy-based evaluation of collateral ligaments was conducted for the first time. We first harvested the ligaments from ACL-transected, contralateral (CL), and intact rabbit knees and subjected them to Raman spectroscopic probing. Five mechanical properties (cross-sectional area, hysteresis at 2 Hz, dynamic modulus at 2 Hz, equilibrium modulus, and structural stiffness) were then measured from the ligament samples using tensile stress-relaxation testing and sinusoidal loading. The relationship between the spectra and mechanical properties of the ligament samples were then investigated using partial least squares (PLS) regression.^1,21,28^ We hypothesised that Raman spectroscopy can estimate the functional properties of ligament from both healthy and injured knee joints. Thus, Raman spectroscopy has the potential to be adapted as a quantitative tool in clinical arthroscopy, providing accurate assessments of tissue structural integrity and leading to improved treatment outcomes. In this study, we develop models that relate Raman spectral data with the biomechanical properties (cross-sectional area, hysteresis at 2 Hz, dynamic modulus at 2 Hz, equilibrium modulus, and structural stiffness) of collateral ligaments.

## Materials and Methods

### Sample Preparation

All experiments and procedures involving animals were approved by the Animal Care Committee at the University of Calgary. The experiments were performed in accordance with the guidelines of the Canadian Council on Animal Care. Skeletally mature female New Zealand White rabbits (strain 052 CR, *N* = 10, 12 months old, 4.8 ± 0.08 kg) were purchased from Charles River Laboratories, Inc. (Saint-Constant, Quebec, Canada). These rabbits were administered subcutaneous acepromazine maleate (1 mg/kg bodyweight, AceVet, Vétoquinol, Inc., Lavaltrie, Quebec) and subcutaneous hydromorphone (0.15 mg/kg bodyweight, HYDROmorphone Hydrochloride Injection USP, Sandoz Canada, Inc., Boucherville, Quebec) to induce anesthesia and were placed under deep surgical anesthesia after 30 min using medical oxygen (1 L/min) with 5% isoflurane (Fresenius Kabi, Inc., Richmond Hill, Ontario, Canada). Surgical anesthesia was maintained with 1–2% isoflurane in medical oxygen while the heart rate and oxygen saturation were monitored with a SurgiVet pulse oximeter (Smiths Medical PM, Inc., Waukesha, Wisconsin, USA). Unilateral ACL transection (ACLT) surgery was performed to artificially induce trauma to either the left or right knee while the CL knee was left intact.

Animals were anesthetised with the procedure as described above, and euthanised at 8 weeks (*N* = 6 animals, 12 knees) post-injury. After euthanisation, the injured (ACLT) joints and the corresponding CL joints were resected from the animals. A separate group of control (CNTRL) animals (*n* = 4 animals, 8 knees) were left unoperated until 8 weeks when the animals were euthanised, and samples were resected. The medial collateral ligaments (MCL) and the lateral collateral ligaments (LCL) were obtained from each knee. ACLT surgery caused fibrosis of the synovial capsule and one LCL from an ACLT knee was damaged and lost during sample preparation. The CNTRL (*n* = 16), ACLT (*n* = 11), and CL (*n* = 12) ligament samples were pooled together for analysis.

### Raman Spectroscopy

**Figure 1:**
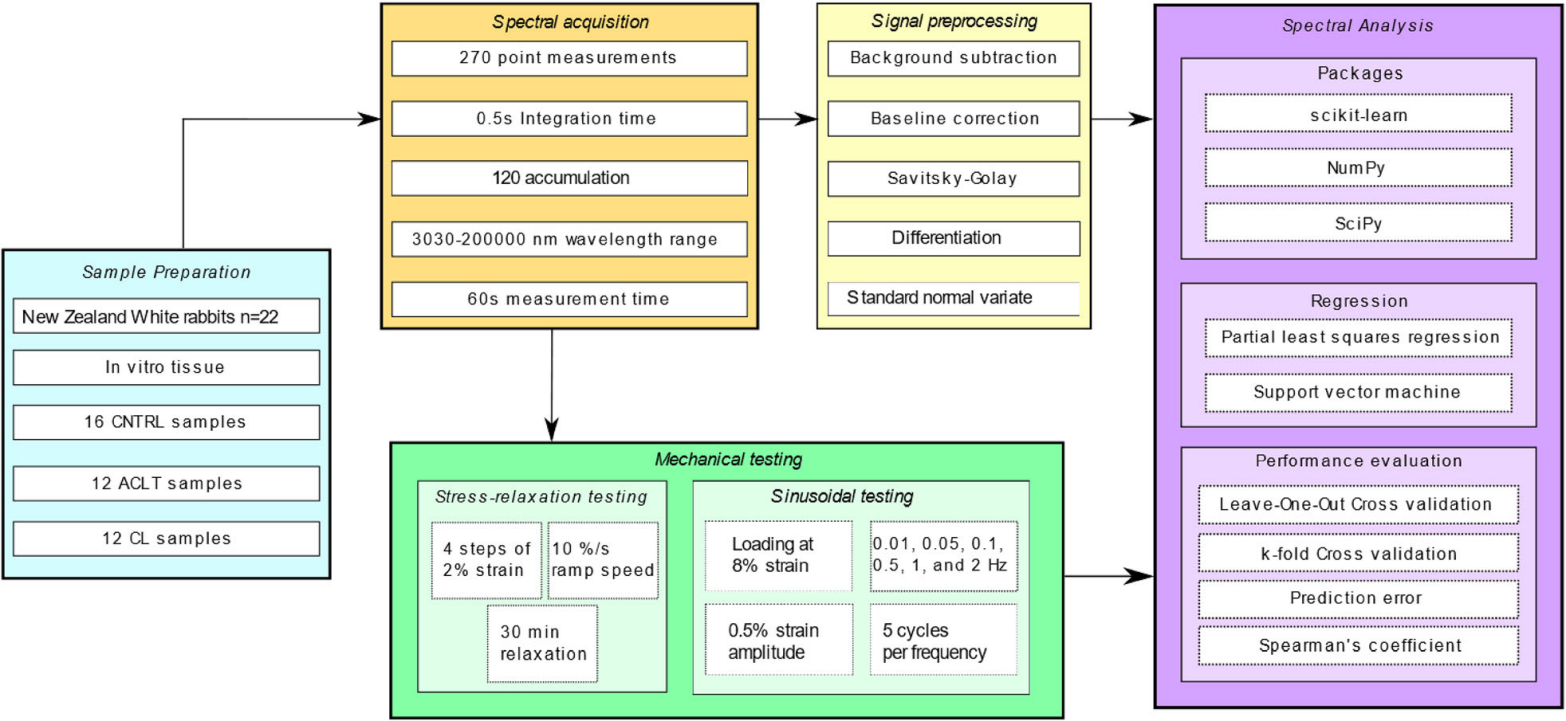
Workflow diagram.

Prior to spectroscopic measurements, the samples were thawed at room temperature. Raman spectroscopy was performed at three equidistant points (labelled A, B and C) along the ligament length using a Raman Imaging Microscope (DXR2xi, Thermo Fisher Scientific, Madison, Wisconsin, USA) in the wavelength range of 3030-200000 nm (wavenumber 50–3300 cm^−1^). Each point was measured three times and averaged to obtain the final Raman spectrum representative of that point. In total, 351 spectra were obtained from 39 specimens. The spectral acquisition parameters (Fig. 1) used were 0.5 s integration time, 120 accumulation (i.e., the number of signals collected and averaged by the charged coupled device), and 1 min total measurement time.

### Mechanical Testing

After spectroscopic measurement, the samples were stored overnight at + 4 °C in phosphate-buffered saline containing protease inhibitors to minimize sample degradation and mechanical testing was performed on the following day. Previously, it has been shown that overnight fridge storage does not significantly impact the biomechanical properties of ligaments and tendon.^22^ As such, overnight storage was deemed more practical than refreezing prior to mechanical testing. To assess the functional integrity of the ligaments, their biomechanical properties were measured via tensile testing using Biomomentum Mach-1 v500csst (Biomomentum, Inc., Laval, Quebec, Canada). A 250 N single axis load cell (Model MA292, Biomomentum, Inc., Laval, Quebec, Canada) and clamp grips for attaching the sample were used. The clamp screws were tightened with a moment of 2 Nm using a moment meter tool, ensuring adequate and constant clamping for all samples. During extensive pilot testing, it was verified that this sample clamping with sandpaper on the clamp surfaces prevented slipping. The cross-sectional area of each ligament was precisely determined using microtomography imaging (Skyscan 1172, Bruker, USA) and ImageJ software. The pixel size of the image was 34.72 *μ*m, and the area was determined by manually segmenting the cross-section from the image. The area was determined at three locations (middle, middle+ − 0.1 mm) and averaged. The testing protocol (Fig. 1) involved a stress-relaxation test and a sinusoidal test for determining the elastic and viscoelastic properties of the tissue. The stress-relaxation test involved four consecutive steps of 2% strain (steps at 2, 4, 6, and 8% strain), with ramp speed of 10%/s, and 30 min of relaxation at each step. Sinusoidal loading was then performed at 8% strain with frequencies at 0.01, 0.05, 0.1, 0.5, 1, and 2 Hz, using a nominal strain amplitude of 0.5%, and 5 cycles per frequency. Five properties were estimated from these measurements: cross-sectional area, hysteresis at 2 Hz, dynamic modulus at 2 Hz, equilibrium modulus, and structural stiffness (Table 1).

**Table 1:** Description, number of samples, standard deviation, and mean value of mechanical target variables in this study.

Variable	*N*	Mean	Std	Description
Area (mm^2^)	39	5.70	2.70	Cross-sectional area of the sample
Hysteresis loss (%)	36	27.46	6.20	Energy dissipation at 2 Hz loading
Dynamic modulus (MPa)	36	102.74	82.29	Stress–strain ratio at 2 Hz loading
Equilibrium modulus (MPa)	39	38.61	33.94	Modulus of the linear region of the stress–strain relationship
Stiffness (N/mm)	39	28.59	18.06	Structural stiffness

### Spectral Preprocessing

Upon collecting the spectra, the quality of the data was assessed. Anomalies were present in the spectral data due to noise, cosmic rays, fluorescence, or spectral contamination.^9^ Background fluorescence heavily interfered with the spectra of interest, and therefore baseline correction of the raw data was required. Once relieved of background interference, the Raman spectra in the region from 750 to 1800 cm^−1^ were smoothed with a 3rd order polynomial Savitzky–Golay filter. A total of 32 preprocessed spectra for each measurement point were generated with varying model parameters including the filter window size (13.49 to 175.32 nm), derivative orders (0, 1st derivative, and 2nd derivative), and normalization with standard normal variate (SNV) preprocessing (with or without).

### Data Analysis

**Figure 2:**
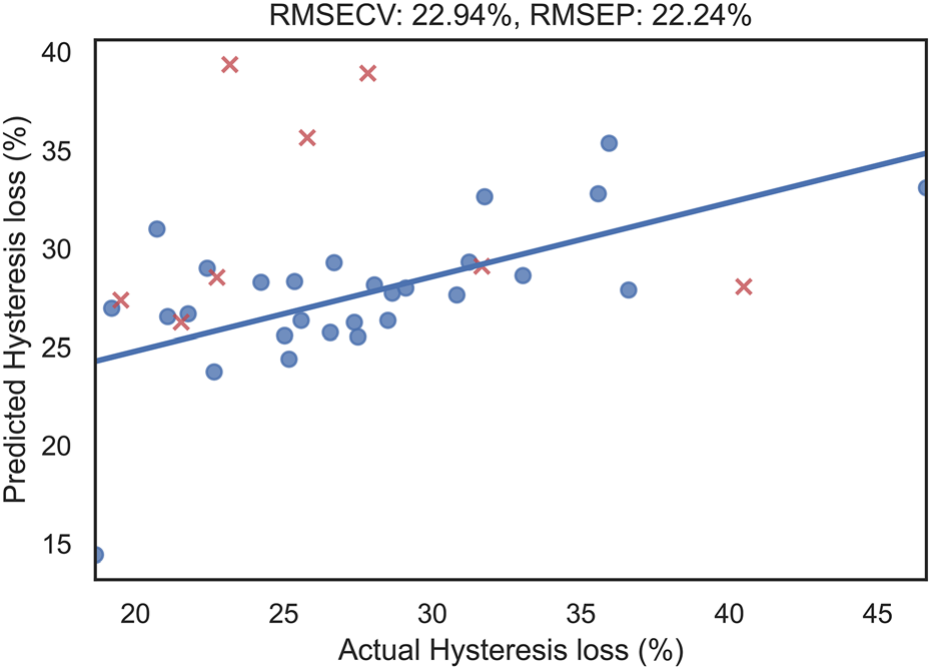
Actual vs. Predicted hysteresis loss from training (blue dot) and test (red cross) data.

Data for all ligament samples, including Raman spectra and the corresponding biomechanical properties, were pooled together for analysis. Multivariate regression analysis of the spectral data was performed in Python using the following packages: NumPy (version: 1.19.5), SciPy (version: 1.6.0), and scikit-learn (version: 0.24.1). The regression models employed PLS and support vector machines (SVM) for correlating the spectra with the biomechanical data, and the performance metrics of the two algorithms were compared. The number of PLS components is shown in Table 4. The PLS components, commonly known as principal components, refer to the most important vectors in the dataset. A grid search was used to select the number of components for PLS as well as tune the hyperparameters of SVM (kernel function, polynomial degree, kernel coefficient, and regularization parameter). Leave-One-Out and stratified *k*-fold cross-validation was performed with 28 and 5 iterations, respectively. The tolerance of the PLS and SVM models’ convergence criteria were 10^−6^ and 10^−3^, respectively. Each of the 32 sets of preprocessed spectra represented a 39 × 545 matrix, i.e., each set consists of spectra for the 39 samples. A separate regression model was developed for each of the five reference biomechanical properties. In order to obtain the best performing model for each target variable, the 32 preprocessed spectra were evaluated independently (Supplementary Fig. S1). The data was split into training and test sets using the pseudorandom shuffle function. In order to produce optimal performance metrics, the training-test split shown in Table 1 was sorted manually. The performance of each model (Fig. 2; Supplementary Figs. S2, S3, S4 and S5) was evaluated using root-mean-square error (RMSE) and the hyperparameters were optimized using grid-search cross validation. Standardization (with or without) using the StandardScaler function along with two different cross validation splitting strategies (Leave-One-Out or 5-fold cross validation) were tested to investigate how these parameters influenced the performance metrics. A total of 160 (32 × 5) RMSE triplets were calculated for training (RMSER), cross-validation (RMSECV) and prediction (RMSEP). This relative error was calculated with the formula below:

$${\mathrm{RMSE}}_{\mathrm{perc}}=\frac{\mathrm{RMSE}\times 100}{{Y}_{\mathrm{max}}-{Y}_{\mathrm{min}}}\%,$$

where RMSE_perc_ is the normalized RMSE, RMSE is the error given in units of the target property, *Y*_max_ is the maximum target value, and *Y*_min_ is the minimum target value.

## Results

**Figure 3:**
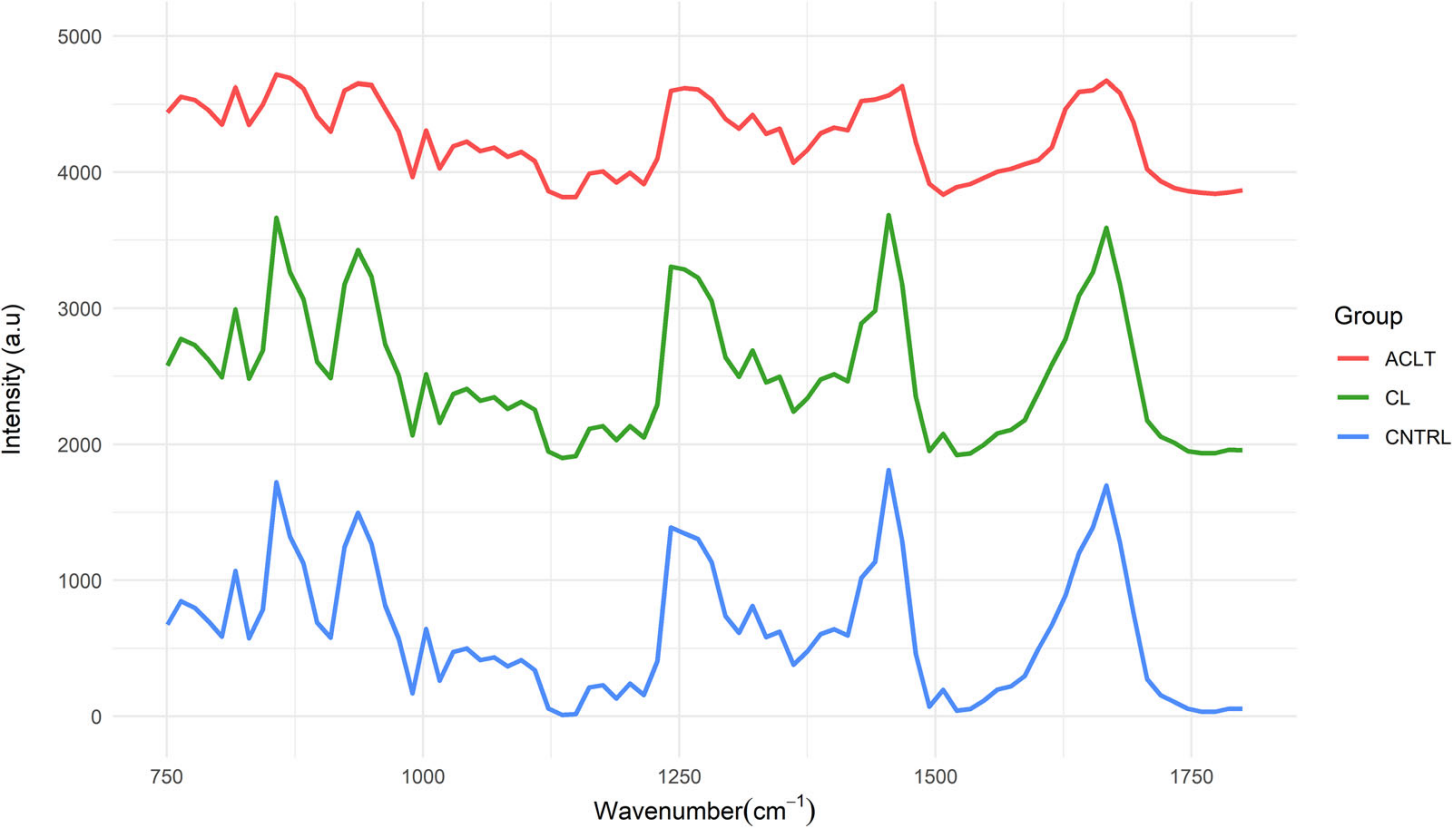
Mean spectra of each group (*ACLT* anterior cruciate ligament transection, *CL* contralateral, *CNTRL* control).

The mean spectra of each sample group (Fig. 3) had similar spectral profiles with the spectra of CNTRL and CL being more similar than the ACLT spectra. Table 4 contains the peak assignments^6,23^ of the mean spectra shown in Fig. 3. Significant differences between the spectra were observed in peaks located around 879, 1257, 1427, and 1668 cm^−1^. The statistical description of the mechanical properties obtained from tensile testing are shown in Table 1. The multivariate model relating the Raman spectra to the ligament cross-sectional area yielded the least error, with RMSEP of 18.15% and Spearman correlation coefficient of 0.57. Among the viscoelastic properties, the least error was observed in the prediction of hysteresis (Fig. 2), with RMSEP of 22.24% and Spearman correlation coefficient of 0.48. Table 2 shows the performance metrics of all models.

**Table 2:** Performance metrics of best pipelines for preprocessed Raman spectra obtained from rabbit ligament samples.

Variable	Validation set					Test set				
	RMSECV	RMSECV (%)	Mean	Std	*ρ*	RMSEP	RMSEP (%)	Mean	Std	*ρ*
Area (mm^2^)	1.82	15.78	1.76	0.07	0.83	2.09	18.15	2.93	0.28	0.57
Hysteresis loss (%)	6.42	22.94	4.88	0.30	0.23	6.22	22.24	12.02	3.06	0.48
Dynamic modulus (MPa)	63.18	19.41	59.59	4.62	0.17	88.42	27.16	134.68	41.55	0.17
Equilibrium modulus (MPa)	24.88	18.54	1.36	0.07	0.12	39.60	29.51	1.47	0.52	0.17
Stiffness (N/mm)	16.24	28.24	16.20	0.60	0.09	17.48	30.39	23.67	4.48	-0.05

*RMSECV* cross validated error, *RMSECV* (*%*) normalized cross validated error, *RMSEP* prediction error, *RMSEP* (*%*) normalized prediction error, *ρ* Spearman correlation coefficient

The best multivariate method for relating the spectra to each target variable varied slightly depending on preprocessing pipeline and cross validation parameters (Table 3). While SVM has been shown to be an effective algorithm for regression and classification in spectral analysis,^3,11,21^ PLS greatly outperformed SVM in direct comparisons. Thus, PLS regression was used for all mechanical target variables. The optimal pre-processing pipeline, PLS model parameter (that is, number of PLS components), and cross-validation strategy for each target variable are highlighted in Table 3.

**Table 3:** Details of best performing pipelines for each mechanical target variable.

Variable	Preprocessing steps			Regression analysis approach			PLS components (*n*)
	Derivative order	Filter window length (nm)	SNV normalization	Standardization	Cross-validation splitting strategy	Model	
Area (mm^2^)	0	59.72	No	No	Leave-One-Out	PLS	2
Hysteresis loss (%)	1	175.32	No	No	Stratified *k*-fold	PLS	1
Dynamic modulus (MPa)	1	59.72	Yes	Yes	Leave-One-Out	PLS	2
Equilibrium modulus (MPa)	1	59.72	Yes	No	Leave-One-Out	PLS	2
Stiffness (N/mm)	1	59.72	Yes	No	Leave-One-Out	PLS	2

All raw spectra were smoothed with a Savitzky–Golay filter with 3rd order polynomial

## Discussion

This study investigated, for the first time, the potential of Raman spectroscopy for estimating the biomechanical properties of healthy and pathological ligaments. The rabbit medial and LCLs were predominantly characterised by spectral peaks located at 879, 950, 1257, 1463, and 1662 cm^−1^ which correspond to proline/hydroxyproline, hydroxyapatite, amide III, glycosaminoglycans and amide I,^6,23^ respectively (Table 4). These assignments are consistent with the fact that glycosaminoglycans, collagen, and other proteins dominate the composition of the solid matrix of ligaments.^15^ The injured (ACLT) and the uninjured ligaments (CL and CNTRL) had similar mean Raman spectra with larger intensity differences observed at the aforementioned peaks (Fig. 3). This difference in intensity may indicate direct reduction in the specific molecule associated with the peak. Figure 3 suggests that this peak reduction is associated with tissue damaged as seen with the ACLT ligaments (red) spectra which exhibit reduced peaks. Due to prior success of near-infrared spectroscopy (NIRS) for predicting structural and compositional properties of connective tissue,^20,28,31^ it is plausible that Raman spectroscopy can yield complementary information to NIRS-based evaluations. Quantitative spectroscopic methods are sensitive to molecular bonds that characterise the underlying chemical structure of tissues^4^ which, in turn, affect the biomechanical properties of ligaments. Hence, it was proposed that Raman spectroscopy, combined with PLS regression, can be used to quantitatively evaluate the structural integrity of ligaments.

**Table 4:** Raman peak assignments^6,23^.

Raman peak (cm^−1^)	Assignment	Molecule
879	$v$(C–C)	Collagen
950	$v$(PO_4_)	Hydroxyapatite
1257	Amide III	Proteins
1463	${v}_{\mathrm{s}}$(COO^−^)	Glycosaminoglycans
1662	Amide I $v$(C–C)	Proteins

The capacity of Raman spectroscopy to estimate ligament properties was best demonstrated in the prediction of tissue cross-sectional area, with a prediction error of 18.15% and Spearman correlation coefficient of 0.57. Among the viscoelastic properties of the ligament samples, the best performing model (based on prediction error) for estimating viscoelastic behaviour of ligament from Raman spectral data was observed for hysteresis, which exhibited the lowest prediction error (RMSEP = 22.24%). Moderate errors were observed in the prediction of the dynamic modulus and equilibrium modulus (27.16 and 29.51%, respectively). It is possible that dynamic and equilibrium moduli models trained with a greater sample size would achieve better results. Limited biological variability in the current dataset could in the future be mitigated through obtaining samples with a good spread of expected moduli values such as varying degrees of degeneration or tears. Raman spectroscopy is sensitive to changes in molecular composition which are affected by collagen network damage and other degenerative changes.^7,24^ Hence, it was postulated that Raman spectroscopy may also yield useful information on the structural and functional properties of ligaments. Collagen is the major extracellular matrix (ECM) constituent in connective tissue and plays a crucial role in their functional properties. It is likely that the relationship observed between the Raman spectra and ligament properties, encapsulated within the PLS models, is due to light scattering by the collagen fibers, which are largely responsible for the viscoelasticity of ligaments. It is worth noting that there are other ECM proteins that work in conjunction with collagen in maintaining the structural integrity of ligaments such as elastin, fibrillin, biglycan, and decorin among others.

There are three key limitations of this study. Firstly, the sample size (*n* = 39 collateral ligaments) was relatively small. While this was the case in this study, due to cost and ethical requirements, this study served as a proof-of-concept that Raman spectroscopy is capable of estimating the properties of ligaments in health and disease. Secondly, findings from *ex vivo* rabbit ligaments may not directly translate into an arthroscopic procedure involving live human patients. Thus, there is a need to evaluate and validate the findings in this study using human samples. Finally, there is not sufficient data to compare Raman spectroscopy with NIRS-based evaluations of ligaments in a meaningful way. The best fitting model for all the biomechanics estimators was PLS, which is suitable for small sample sizes.^13,14,21^ Since PLS is considered the gold standard analytical method for spectral analysis,^21^ adopting PLS regression models was appropriate for the purpose of this study. The cartilage of the same group of rabbits (divided and labelled as control, ACLT and CL groups) were investigated in a previous study^3^ in order to classify the samples into healthy and pathological cartilage using NIRS. Moreover, Raman spectroscopy has been demonstrated to be an effective tool for evaluating cartilage,^19,24^ providing critical information about the biochemical changes that occur at the onset and progression of OA. However, due to the relatively small dataset in this study, classification analysis of the ligaments was not a plausible approach for tissue characterisation.

More extended study may be required to translate the outcomes of this study to arthroscopic assessment of ligament integrity in human patients. Previously, it has been shown that quasi-static ligament properties can be estimated using NIRS with a relatively low error.^28^ However, an ultimate tensile test until tissue failure, which will yield quasi-static material properties, was not performed in this study as these ligaments were needed for further processing after biomechanical testing. As such, future studies to comprehensively compare Raman and NIRS spectroscopy should evaluate the prediction performance of both techniques on quasi-static material properties. While these limitations do not affect the outcomes and conclusions of this study, they should provide guidance for future investigations. More so, these studies could provide a guide for combining the diagnostic power of both techniques in a multi-modal approach for ligament characterisation.

The characterisation of biomechanical properties using spectroscopic and regression methods could greatly improve outcomes of arthroscopic repair surgery.^2,20,26^ By probing the knee with a minimally invasive protocol, viscoelastic and quasi-static properties could be measured in real-time during arthroscopy. In order to design a fully integrated system with an arthroscopic probe that can estimate biomechanical properties of human tissues, a real-time interface must be implemented. Such a system must be capable of providing orthopaedic surgeons with the minimal information necessary to make an accurate evaluation of the structural integrity of knee ligaments. Performing and preprocessing measurements is a rapid process however, whether a linear or a non-linear model should be adopted for this application will present a trade-off between computation time and error. Computational efficiency can be resolved with the proper hardware but, as the results of this study suggest, PLS regression can predict certain parameters (cross-sectional area and hysteresis) accurately while not so much for others (dynamic and equilibrium moduli). Further investigation is required to determine the best approach to adopt when designing a system capable of unsupervised learning for making real-time predictions on human subjects.

The results of this proof-of-concept study provide an indication of the predictive potential of Raman spectroscopy as this field of research is still in its infancy. Future studies must investigate more complete datasets which include quasi-static material properties for comparison with NIRS. Another study that would aid in mapping the structural integrity of the knee is to investigate the capacity of Raman spectroscopy for estimating biochemical properties of the tissue, such as cellularity, water content, proteoglycan content, elastin content, collagen content, *etc*.

By developing a supervised regression model capable of determining the relationship between the spectral data and viscoelastic properties, this technique can potentially improve assessments of connective tissues during arthroscopy. Previously, it has been shown that NIRS has the potential for real-time evaluation of the structural integrity of connective tissues during arthroscopic procedures. Furthermore, Raman spectroscopy produces complementary information using a nearly identical operating principle and form-factor. While Raman spectroscopy has been used for cartilage analysis, this study is the first demonstration of its potential for evaluating the structural integrity of collateral ligaments. The combination of Raman spectroscopy with other spectroscopic techniques, such as NIRS, could augment conventional arthroscopic approaches with a rapid non-destructive evaluation of the ligaments and other connective tissue of the knee. This is because of the subjective nature of traditional arthroscopy which relies on visual evaluation (camera) and manual palpation (arthroscopic hook) as the primary tools for assessing tissue integrity. The implementation of a quantitative interpretation of the compositional and structural integrity of ligaments into arthroscopy will vastly improve diagnostic outcomes, patient quality of life, and our understanding of the physiological changes that take place in diseased joints.
